# Identification of p.Arg205Cys in *CASR* in an autosomal dominant hypocalcaemia type 1 pedigree: A case report

**DOI:** 10.1097/MD.0000000000026443

**Published:** 2021-06-25

**Authors:** Yubing Ji, Chunyang Kang, Jiajun Chen, Lei Zhang

**Affiliations:** Department of Neurology, China-Japan Union Hospital of Jilin University, Changchun, Jilin, China.

**Keywords:** calcium-sensing receptors, hypercalciuric, hypocalcemia, mutation

## Abstract

**Rationale:**

Autosomal dominant hypocalcaemia type 1 (ADH1) is a genetic disease characterized by benign hypocalcemia, inappropriately low parathyroid hormone levels and mostly hypercalciuria. It is caused by the activating mutations of the calcium-sensing receptor gene (*CASR*), which produces a left-shift in the set point for extracellular calcium.

**Patient concerns:**

A 50-year-old man presenting with muscle spasms was admitted into the hospital. He has a positive familial history for hypocalcemia. Auxiliary examinations demonstrated hypocalcemia, hyperphosphatemia, normal parathyroid hormone level and nephrolithiasis. A missense heterozygous variant in *CASR*, c 613C > T (p. Arg205Cys) which has been reported in a familial hypocalciuric hypercalcemia type 1 patient was found in the patient's genotype. It is the first time that this variant is found associating with ADH1. The variant is predicted vicious by softwares and cosegregates with ADH1 in this pedigree. *CASR* Arg205Cys was deduced to be the genetic cause of ADH1 in the family.

**Diagnosis:**

The patient was diagnosed with ADH1 clinically and genetically.

**Interventions:**

Oral calcitriol, calcium and hydrochlorothiazide were prescribed to the patient.

**Outcomes:**

After the treatments for 1 week, the patient's symptom was improved and the re-examination revealed serum calcium in the normal range. A 3-month follow-up showed his symptom was mostly relieved.

**Lessons:**

The variant of *CASR* Arg205Cys, responsible for ADH1 in this family, broadened the genetic spectrum of ADH1. Further and more studies are required to evaluate the correlation between genotype and phenotype in ADH1 patients.

## 1 Introduction

Autosomal dominant hypocalcaemia 1 (ADH1, MIM#601198) is characterized by varying degrees of hypocalcemia, a tendency towards hyperphosphatemia, low-to-normal serum parathyroid hormone (PTH) levels and relative hypercalciuria.^[1]^ Most patients with ADH1 are known to have activating mutations of the calcium-sensing receptor gene (*CASR,* MIM#601199).^[2]^ The protein of calcium-sensing receptor (CASR) is a G protein-coupled cell surface receptor and mainly expressed in parathyroid chief cells, calcitonin-producing thyroid cells, bone cells and renal tubular cells. It is the key sensor for extracellular calcium and controls parathyroid secretions.^[3]^ Here, we report the clinical and laboratory features of an ADH1 pedigree as well as an activating variant of *CASR* Arg205Cys in this pedigree.

## 2 Clinical data

**Figure 1:**
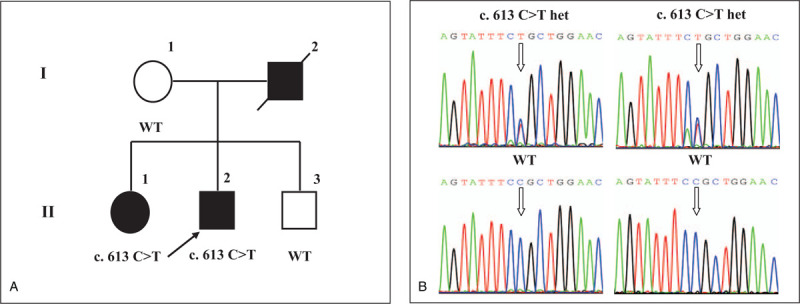
Detection of *CASR* Arg205Cys in the family with ADH1. (A). Pedigree of the family with ADH1. The proband (II-2) is indicated by an arrow. Filled symbols, individuals with hypocalcaemia; open symbols, unaffected individuals; slash, deceased; squares, male; circles, female individual. (B). A heterozygous C-to-T transition at the 613 nucleotide was identified in the proband and his sister by Sanger DNA sequencing and confirmed cosegregating with hypocalcemia. The c.613C > T mutation produces an amino acid change from arginine to cystine at codon 205 (Arg205Cys). Arrow denotes described the variant. The left and right upper figures show the variant of Arg205Cys identified in the proband's and his elder sister's genotype. The left and right lower figures show that his mother and brother were wild type. WT = wild type.

The proband, a 50-year-old male (II-2 in Fig. 1A and Table 1), presented with muscle spasms since the age of 5 years old and they involved his hands, feet, abdomen and chest. He was diagnosed with hypocalcemia when he was 9 years old. The symptom was relieved during summer when he did not need to take any medicine. But in winter, especially before and during snowing, muscle spasms were so severe that he had to intravenously inject calcium intermittently. In recent 5 years, he felt the symptom was more severe than before, since in winter he had to inject calcium for a longer time. He had taken calcium and calcitriol irregularly in the past. When he was 40 years old, he had nephrolithiasis once.

**Table 1:** Biochemical characteristics of family members.

Pedigree No.	Age/Sex	*CASR*	Ca (mmol/L)	P (mmol/L)	Mg (mmol/L)	PTH (pg/ml)	Urine Ca (mmol/24h)
Reference range	–	–	2.1–2.55	0.81–1.45	0.7–1	15–68.3	1–8.8
I–1	78/F	WT	2.36	0.98	0.85	29.6	2.14
II–1	53/F	Arg205Cys	1.98	1.46	0.75	18.4	5.04
II–2	50/M	Arg205Cys	1.97	1.57	0.72	24.3	4.86
After treatment	–	–	2.13	1.73	0.88	–	1.16
II–3	52/M	WT	2.24	1.32	0.82	32.6	1.89

*CASR* = calcium-sensing receptor gene, WT = wild type.

He has a family history of hypocalcaemia in his father and his elder sister (Fig. 1A). His father (I-2 in Fig. 1A) was said to have the symptom of muscle spasms in his 30's. But the symptom only lasted for about 5 years and relieved spontaneously. He had never done any blood test or treatment. He died of chronic obstructive pulmonary disease at the age of 69. The proband's elder sister (II-1 in Fig. 1A) was now 53 years old and has been found slightly hypocalciumia with serum calcium 2.05 during her pregnancy, but she did not have any symptom and never gave any treatment.

This study has been reviewed and approved by the Ethics Committee of the China-Japan Union Hospital of Jilin University. The patient and his family have provided written informed consent to the participation in the study and authorized to publish the study in accordance with the Declaration of Helsinki.

### 2.1 Physical examination

The proband was normal status and normal morphology of face, limbs and fingers. He did not demonstrate any paresthesia. Chvostek and Trousseau signs were negative. Muscular contractions in the hands and arms could be observed.

### 2.2 Auxiliary examinations

On admission, he was under neither calcium nor calcitriol treatment. The results of laboratory examinations and reference ranges were included in Table 1. They showed hypocalcemia, hyperphosphatemia, normal PTH and urinary calcium, while magnesium was at the lowest level. Serum creatinine levels and other electrolyte levels such as sodium, potassium or chloride were in the normal range.

There was no calcification seen on a brain CT. Serial renal tract ultrasounds demonstrated stable grade 1 nephrocalcinosis.

### 2.3 Genetic testing

Venous blood samples were collected from the proband and his family members. Genomic DNA was extracted using a standard method and targeted NGS was performed on the proband's DNA for 574 genes currently related to inherited metabolic disorders in Beijing Kanso Gene Corporation. A heterozygous variant of c.613 C > T in the exon 4 of *CASR* was found. This sequence change resulted in a missense substitution of arginine with cysteine at codon 205 (p.Arg205Cys) which was located in the extracellular domain of the CASR protein. This genetic result was further confirmed by direct Sanger sequencing (Fig. 1B) and tested in other individual of his family. His mother and his brother were wild type genotypes and his elder sister who was hypocalciumia and asymmetric also carried *CASR* Arg205Cys (Fig. 1A and B). This confirmed the cosegregation of the variant with hypocalcemia in the pedigree. The arginine residue is highly conserved and there is a large physicochemical difference between arginine and cysteine. This variant is present in population databases (rs775751453) but its frequency is very low (ExAC 0.01%). According to American College of Medical Genetics and Genomics standards and guidelines, this variant is classified as “pathogenic”. Furthermore, according to the prediction tools PolyPhen2 and SIFT, this mutation is characterized as “possibly damaging” and “deleterious,” respectively. Thus, the variant of *CASR* Arg205Cys is likely to be pathogenic.

### 2.4 Treatment and outcome

The proband was treated with calcitriol 0.25 μg and calcium 600 mg daily. At the same time, hydrochlorothiazide (0.5 mg/kg/day) was additionally administered to decrease urinary calcium excretion. One week later the repeated blood test revealed that the serum calcium was increased to the lowest range and the phosphate and magnesium level were also increased (Table 1). Serum potassium was 2.98 mmol/L (3.5–5.5 mmol/L) and oral potassium was prescribed to him. A 24-hour urine calcium while on treatment was 1.16 mmol/day, much lower than before. No neurological symptom was present. The patient is currently being followed up for 3 months and the doses are regulated according to blood monitoring, urine tests and renal ultrasonography.

## 3 Discussion

Autosomal dominant hypocalcaemia (ADH) refers to hypocalcemia caused by the defects of *CASR*. Up to now, 2 types of ADH have been identified: autosomal dominant hypocalcemia types 1 and 2 (ADH1 and ADH2) and they are caused by germline gain-of-function mutations of *CASR* and its signaling partner, the G-protein subunit α 11 respectively with the former more common.^[4]^ Classically, ADH1 is characterized by hypocalcemia with hyperphosphatemia, normal or low circulating PTH concentrations, and a relative or absolute hypercalciuria. However, the presentation of hypocalcemia varies from mild asymptomatic to severe ones such as seizures, paraesthesias, carpopedal spasms or a febrile seizures, sometimes with severe outcome manifesting with tetany or laryngospasm.^[5]^ Patients with ADH1 may also present with complications due to hypercalciuria, like renal stones, nephrocalcinosis, renal impairment and basal ganglia calcifications.^[6]^ Interestingly considerable variation in the clinical presentation of 1 hypocalcemic family members can be observed. In a family with ADH1 caused by *CASR* Gly604Leu, 1 family member suffered from severe muscle cramps and nephrocalcinosis since childhood, whereas 2 other hypocalcaemic members remained asymptomatic.^[7]^ In this study, the proband presented with the typical muscle spasms, and hypocalcemia, hyperphosphatemia, normal PTH and urinary calcium. While his father suffered from hypocalcemic symptoms in his 40's lasting for only several years, while his elder sister, who also harbers the same *CASR* variant and hypocalciumia, does not show any related symptoms.

The protein of CASR is a cell surface-expressed G protein-coupled receptor. The mature human CASR protein is composed of 1078 amino acids and consists of 3 functionally significant domains-a large N-terminal extracellular domain (1–612 amino acids), a 7 transmembrane domains (613–867 amino acids), and a long C-terminal intracellular domain (868–1078 amino acids).^[8]^ CASR is abundantly expressed in the parathyroid glands and kidney, where it plays a key role in the maintenance of Ca^2+^ homeostasis through Ca^2+^-mediated regulation of PTH release and renal tubular Ca^2+^ reabsorption. Generally, when the calcium concentration in the blood is lowered, the secretion of PTH is increased, which then increases the reabsorption of calcium in the kidneys, ultimately leading to elevated blood calcium levels.^[9]^ The importance of the CASR in the regulation of Ca^2+^ has been highlighted by the identification of loss-of-function *CASR* mutations that give rise to familial hypocalciuric hypercalcemia type 1 and neonatal severe hyperparathyroidism, as well as by gain-of-function *CASR* mutations that cause ADH1.^[4]^ Activating *CASR* mutations increase the sensitivity of the receptor to extracellular Ca^2+^, resulting in low or inappropriately normal PTH levels and reduced renal Ca^2+^ reabsorption. Most reported mutations causing ADH1 are reported to be heterozygous missense ones.^[10]^ Here in this pedigree, ADH1 is caused by the variant of *CASR* Arg205Cys. It is located at the extracellular domain and is essential for ligand binding. It has been suggested that mutations in this region affect the three-dimensional structure of the receptor, resulting in a “more sensitive” conformational state.^[11]^ However this variant has previously been reported in a familial hypocalciuric hypercalcemia type 1 patient without detailed clinical and genetic information.^[12]^ It is the first time that this variant is found associating with ADH1. Indeed such a different result waits more clinical and functional studies to prove its pathogenicity.

Currently, ADH1 patients receive treatment with conventional medication, such as calcium and vitamin D. But this treatment should be reserved due to the risk of hypercalciuria and severe complications such as nephrocalcinosis, nephrolithiasis and renal impairment.^[13]^ Therefore, hydrochlorothiazide (0.5–2.0 mg/kg/day) should be administered alongside, leading to the increased reabsorption of calcium in the renal tubules. At the same time, hypomagnesemia reduces parathyroid secretions and vitamin D synthesis, and it results in unresponsiveness or hyporesponsiveness in the organs on which PTH acts.^[14]^ So hypomagnesemia should be corrected preferentially. PTH (1–34) could also correct urinary calcium excretion and prevent renal complications in ADH1 patients.^[15]^ Calcilytics resulted in functional correction of the altered CASR with activating mutation in vitro.^[16,17]^ In the present case, renal stones were observed on the patient's admission. So hydrochlorothiazide with minimal calcium and calcitriol was administrated to prevent severe hypercalciuria. Blood and urine tests along with renal ultrasonography were regularly performed during the patient's follow-up for the dosage monitoring and modification. Finally the treatment has gained a satisfactory result both in symptoms and laboratory tests.

In summary, we report a family with ADH1 that carries a *CASR* Arg205Cys variant which has been reported in a FHH1 patient. It is the first time that this variant is found associating with ADH1. More clinical and functional studies are necessary to prove its pathogenicity. Accurate diagnostic confirmation of ADH1 with genetic testing is important to facilitate proper management, screening, genetic counselling and to provide rational treatment.

## Author contributions

**Methodology:** Yubing Ji, Chunyang Kang.

**Supervision:** Lei Zhang.

**Writing – original draft:** Yubing Ji, Chunyang Kang.

**Writing – review & editing:** Jiajun Chen.
